# Effect of Single Nucleotide Polymorphism Rs189037 in ATM Gene on Risk of Lung Cancer in Chinese: A Case-Control Study

**DOI:** 10.1371/journal.pone.0115845

**Published:** 2014-12-26

**Authors:** Jing Liu, Xiaobo Wang, Yangwu Ren, Xuelian Li, Xichen Zhang, Baosen Zhou

**Affiliations:** 1 Department of Epidemiology, School of Public Health, China Medical University, Shenyang, 110001, Liaoning Province, China; 2 Key Laboratory of Cancer Etiology and Intervention, University of Liaoning Province, Shenyang, China; 3 Clinical Medicine of Seven System, China Medical University, Shenyang, China; University of Algarve, Portugal

## Abstract

**Background:**

Accumulated evidence has indicated that ataxia-telangiectasia mutated (ATM) gene polymorphisms are closely related to lung cancer. We aimed to explore the prognostic value of rs189037 (G>A), one of ATM single nucleotide polymorphisms (SNPs), and detect whether it involves in the risk of lung cancer in Chinese Han people.

**Methods:**

In this hospital-based matched case-control study, 852 lung cancer patients and 852 healthy controls have been put into comparison to analyze the association between rs189037 and lung cancer risk in Chinese. The single nucleotide polymorphisms were determined by TaqMan real-time PCR and we used SPSS software to perform the statistical analyses.

**Results:**

Individuals carrying variant AA genotype of rs189037 had higher lung cancer risk (adjusted OR: 1.56) than those carrying GG genotype. After analyzing data respectively from different groups divided by genders and smoking status, we observed that the risk effect of AA genotype on the lung cancer was significant in females, non-smokers and female non-smokers, as well as the risk effect of GA genotype in male smokers. Compared with non-smokers carrying GG genotype, smokers carrying at least one A allele had higher risk of developing lung cancer than those with GG genotype (adjusted OR: 3.52 vs. adjusted OR: 2.53).

**Conclusions:**

This study suggested that rs189037 (G>A) polymorphism is associated with lung cancer risk in Chinese Han population. AA genotype and A allele may be dangerous lung cancer signals in Chinese and make contribution to diagnostic and treatment value.

## Introduction

Lung cancer is one of the most lethal malignancies and remains a leading cause of cancer-related death in many countries. Although smoking is considered to be a common cause of lung cancer, global cancer statistics state that 25% of all lung cancer cases in the world are not due to smoking, including 15% of male lung cancers and 53% female lung cancers [1], which suggests that there are individual variations about lung cancer carcinogens. For Chinese people, the incidence of lung cancer increases by 1.63% per year, 1.30% for men and 2.34% for women [2]. Considering a large proportion of women are still non-smokers, many researchers presume environment and lifestyle are the predisposed causes among females [3]–[5]. However, only a part of individuals exposed to carcinogen developed lung cancer, which implies that there may be genetic factors leading to a higher susceptibility to cancers. Genome-wide association studies (GWAS) suggest that single nucleotide polymorphisms (SNPs) in various genes located in several chromosomes are associated with lung cancer risk [6]–[9].

Ataxia-telangiectasia mutated (ATM) protein is a 370 kDa serine/threonine kinase [10], but only several domains have been clearly detected. ATM is generally believed to remain constant with the cells to keep constitutively expressing, it is suggested to be involved in the DNA Damage Response (DDR) pathway which is triggered by extensive DNA damage [11]. ATM acts as caretaker of the genomic health so that it can be activated by genomic insults [12]. As a core part in DNA damage response, ATM is involved in DNA repair, cell arrest, chromatin remodelling and cell apoptosis [13]. Additionally, it plays a part in oxidative stress and cellular metabolism. Therefore, ATM seems like a handler of cell fate via its major substrates. Linkage analysis detects that ATM gene located on chromosome 11q22–23 transcripts mRNA with a coding sequence of 9168 bp [14], and it is responsible for ataxia telangiectasia, an autosomal recessive inheritance disorder. Epidemiological studies have demonstrated that ATM gene polymorphisms are related to cancer risk including lung cancer, breast cancer, glioma and pancreatic cancer [15].

Numerous Epidemiological statistical researches have drawn a broader picture to produce a relationship between ataxia-telangiectasia mutation and the incidence of various cancer [14], [16]. Exposed to DNA damaging agents, a series of cellular responses and repair pathways in normal cells would be triggered, but the ability of repairing DNA damage in ATM-deficiency cells would be decreased. As several previous studies significantly support the association between ATM signal pathway and lung cancer susceptibility, we did a further research concentrating on ATM gene polymorphism and its association with lung cancer risk. We investigated the distribution of rs189037 genotypes in cases and control groups to confirm whether it contributes to lung cancer risk in Chinese Han population.

## Materials and Methods

### Study subjects

The name of the ethics committee is China Medical University Ethics Committee and we obtained ethics approval. We have uploaded a formal written waiver of ethics approval to your system. All participations provided written consent form, and their family members also signed the consent form. Ethics Committee approved our consent procedure.

In this hospital-based case-control study, we recruited 852 lung cancer patients with histologically confirmed diagnoses. Patients should meet the following criteria:

1. All cases should meet lung cancer diagnostic criteria announced by World Health Organization (W.H.O) in 2004

2. They should be primary lung cancer

3. They have not used chemotherapy or radiation therapy

During the same period, 852 healthy controls with no evidence of lung or other cancers were randomly recruited from a medical examination center in the same hospital. Participants were unrelated ethnic Han Chinese and signed informed consent. Face-to-face interviews of patients and healthy control subjects were conducted by two trained interviewers, collected information including demographic data (name, gender, age, etc.) and smoking status. All of information was entered by Epi-info software after repeated checking and strict coding.

### Genotyping

DNA was extracted from 1 ml samples of whole blood using standard phenol–chloroform methods. Genotyping was performed on an Applied Biosystems 7500 FAST Real-Time PCR System (Foster City, CA, USA) using a TaqMan SNP genotyping assay (Affymetrix Inc., Cleveland, Ohio, USA). Each reaction (10 µl) contained 5 µl TaqMan Genotyping master mix, 0.5 µl primers and probes (Applied Biosystems), 2.5 µl water and 2 µl DNA (15–25 ng/µl). Thermal cycling conditions were 95 cycling conditions d Biosystems), 2.5 n an Applied Biosystems 7500 FASmin. Duplicates of 10% of the samples were re-tested for quality control purposes. To confirm the genotyping results and assess the reproducibility, 10% duplicated samples were sequenced randomly, and these results were 100% concordant.

### Statistical analysis

The chi-squared test was used to examine differences between patients and controls. The Hardy-Weinberg equilibrium was tested using a Pearson chi-squared test. The odds ratio (OR) and its 95% confidence interval (95% CI) were obtained by logistic regression methodology to determine correlations between the rs189037 polymorphism, and the incidence of lung cancer in Chinese. All analyses were performed using SPSS 13.0 software (SPSS, Inc. Chicago, IL, USA), and a P<0.05 was considered to be statistically significant.

## Results

This study comprised 852 lung cancer cases and 852 controls, who were all ethnic Han Chinese.

S1 Table presents the genotype distribution of ATM SNP rs189037 and its associations with lung cancer risk in this Chinese Han population. In the total population, rs189037 was found to be significantly associated with risk of lung cancer. The frequencies of the GG, GA and AA genotypes of rs189037 were 25.5, 53.1, 23.5% in cases and 31.0, 50.9, 18.1% in controls, respectively. Adjusting odd ratios for age, gender and smoking factor, a significantly increased risk of lung cancer was observed among the participants with homozygous variant genotype (AA) (adjusted OR: 1.56, 95% CI: 1.18–2.08), compared with the homozygous wild type (GG). Moreover, similar significant association was observed in female population, compared with those females carrying GG genotype, adjusted OR (95%CI) of the females with AA genotype was 1.75(1.14–2.68), which means rs189037 AA genotype may be a risk factor of Chinese female lung cancer, while no such effect existed among males(P>0.05).

We respectively studied associations between ATM rs189037 and lung cancer risk in non-smokers and smokers. In smoking population (S2 Table), GG, GA and AA genotypes of rs189037 were 25.2%, 52.2%, 22.6% in cases and 31.9%, 47.3%, 20.9% in controls. We found that male smokers carrying rs189037 GA genotype showed an increased risk compared to male smokers carrying homozygous wild type (GG) (adjusted OR: 1.63, 95% CI: 1.04–2.55), but no significant associations could be found in overall smokers or female smokers (P>0.05). In non-smoking population (S3 Table), genotype distribution of rs189037 GG, GA, AA type were 25.9%, 49.6%, 24.5% in cases and 30.6%, 52.7%, 16.8% in controls. After we analyzed data in non-smokers, we found the rs189037 AA genotype carriers showed an increased risk for lung cancer when compared to homozygous wild type (GG) in non-smokers (Adjusted OR: 1.73, 95%CI: 1.18–2.52). Separate analyses showed that the lung cancer risk effect of the AA genotype was significant in female non-smokers (Adjusted OR: 1.86, 95%CI: 1.08–3.21), but no sense in male non-smokers.

As shown in S4 Table, we assumed non-smokers carrying wild type GG genotype of rs189037 as a reference, and we found whether the smokers carry A allele or not, they all had an increased risk of lung cancer. What is more, smokers carrying at least one A allele had a 1.39-fold increased risk of developing lung cancer than smokers carrying GG genotype (adjusted OR: 3.52 vs. adjusted OR: 2.53).

## Discussion

There are not only a great many of but also various kinds of etiologies of lung cancer, however not all are clear now. For Chinese people, the incidence of lung cancer is increasing per year. We did this case-control study to dig out the association between ATM single nucleotide polymorphism (SNP) rs189037 and lung cancer risk in a Chinese population, which supported that genetic factor played an important role in individual lung cancer susceptibility.

In the present study, we selected and genotyped rs189037 in the promoter region of ATM gene located on 11q22–23, there is a statistically significant association between rs189037 and lung cancer risk in this Chinese Han population. The association is consistent with a pathway-based analysis in 2012, Dong J et al. examined 218 SNPs in 50 DNA repair genes in 568 lung cancer survivals and found 6 SNPs associated with lung cancer prognosis, including ATM rs189037, MRE11A rs11020802, ERCC2 rs1799793, MBD4 rs140693, XRCC1 rs25487, and PMS1 rs5742933 [8]. Compared with the homozygous wild type (GG) in our Chinese people, we observed participants with the homozygous variant genotype (AA) of ATM SNP rs189037 contributed to increased lung cancer risk (adjusted OR: 1.56, 95% CI: 1.18–2.08). Several previous studies indicated that individuals carrying AA genotype of rs189037 had higher risk of breast cancer [17] and oral cancer [18], which suggested that variant AA genotype might be a dangerous signal of developing malignances, and Xu L et al. pointed out that wild genotype GG of rs189037 was a protective effect against differentiated thyroid carcinoma(DTC) [19]. We assumed that GG genotype might also be protective gene against lung cancer. In addition to malignant tumor, AA genotype of rs189037 also has negative effects on other diseases, for example, Xiong H et al. indicated that AA type could predict severe radiation pneumonitis in patients with non-small cell lung cancer after definitive radiation therapy [20]. The study of Li et al. showed that both of AA genotype and A allele were associated with an increased risk of idiopathic nonobstructive azoospermia (INOA) [21]. However, there are several studies competing with our research. In the earliest research about the relationship between rs189037 and lung cancer, Kim et al. invited 616 lung cancer patients in Korean population and evaluated several ATM genotypes separately, but no significant association was found between this polymorphism and lung cancer risk (P>0.05) [22]. In 2010, Lo et al. genotyped 9 SNPs in 730 lung cancer patients and 730 healthy controls in Taiwan but didn't find the association between rs189037 and lung cancer risk among all participations(P>0.05) [23]. In 2013, Hsia et al. investigated 358 lung cancer patients and 716 controls in Taiwan, neither did they find the association. They found no genotype frequency difference between lung cancer cases and controls among smokers (P>0.05) [24]. These conflicting results may be due to multiple reasons. Firstly, for the different research population from different areas, the various kinds of environments and lifestyles would influence the lung cancer susceptibility. Secondly, smoking status might modulate lung cancer risk and be a confounder in the association. On the top of this, most of researches were limited by small size population. When public attention has been drawn to risk factors including smoking exposure, indoor environment and lifestyle in Chinese females [5], [25], we should also pay more attention to individual genetic factor. In our study, significant association between rs189037 AA genotype and lung cancer risk was observed in Chinese females (adjusted OR: 1.75, 95% CI: 1.14–2.68), but not in Chinese males. This demonstrated that AA genotype might be a dangerous signal of Chinese female lung cancer.

Considering smoking status, we studied rs189037 in non-smokers and smokers respectively. Interestingly, we are in line with Lo et al. about lung cancer risk in non-smokers [23], AA genotype carriers showed an increased risk when compared to wild type GG genotype in non-smokers (Adjusted OR: 1.73, 95%CI: 1.18–2.52). In further separate analyses, we found that Chinese female non-smokers carrying AA genotype were more likely to develop lung cancer than others carrying GG genotype, which is consistent with the recently published result of another team in our laboratory, they genotyped rs189037 in 487 lung cancer patients and 516 healthy controls. Their study suggested that AA genotype might be a risk factor of lung adenocarcinoma in Chinese female non-smokers without cooking oil fume exposure (adjusted OR: 1.89, 95%CI: 1.03–3.49, P = 0.040) [26]. There was a noteworthy phenomenon in our study, we found no significant association between rs89037 and lung cancer risk in males (P>0.05), but male smokers carrying GA genotype had an increased lung cancer incidence (adjusted OR: 1.63, 95% CI: 1.04–2.55). We observed an increased lung cancer risk in females carrying rs189037 AA genotype (adjusted OR: 1.75, 95% CI: 1.14–2.68), but female smokers with variant genotype were not associated with lung cancer risk(P>0.05). It is a generally acknowledged fact that smoking status largely influences the risk of lung cancer. Regardless of rs189037 genotypes, the risk of lung cancer in smokers was increased (GG type Adjusted OR: 2.53, GA+AA type Adjusted OR: 3.52). Lung cancer risk effect of A allele added to the result so that smokers carrying at least one A allele had a 1.39-fold increased risk of developing lung cancer than GG carries. Tobacco smoke is a complex mixture containing numerous mutagens and carcinogens. SNPs of various genes may participate in affecting dopamine reward mechanisms due to nicotine, carcinogen metabolism and detoxification [27]. At the molecular level, both gene polymorphisms and carcinogens could alter individual capability of DNA repair, cell cycle control and other cellular responses.

Rs189037 is a single nucleotide polymorphism (SNP) located in the promoter region (non-coding region) of ATM gene. Rs189037 polymorphism can't directly influence amino acid coding of ATM protein. So it is possible to participate in splicing, intervention, modification, determination process changing RNA stability and then influences the expression level of ATM protein [17]. To figure out how this polymorphism regulates the ATM mRNA expression, Tie Chen et al. analyzed the sequences around the SNP rs189037 and demonstrated that AP-2a binds to the SNP site to repress ATM transcription [28], according to the bioinformatics analysis by Leask et al. in 1991 [29]. To date, the ATM protein kinase has been straightly considered to be a DNA damage sensor and one of therapeutic targets for cancer [30]. Accumulated evidence has demonstrated that ATM protein is activated immediately in response to DNA double strand breaks (DSB) caused by either genetically programmed or the appearance of selected exogenous factors [31], [32]. With the purpose of rapid and accurate sense and repairing damage in cellular lesion, the signal is generated to recruit ATM kinase to DSB sites and cause the phosphorylation of multiple ATM substrates [32]. Cells have formed complex molecular networks to sense DSBs and coordinate their repair. Individuals who have deficiencies in the abilities above might lead to the cell death, somatic mutations, and carcinogens [31]. ATM promotes the recombination of DSB intermediates and prevents broken DNA ends from chromosome mutation [33]. ATM mutation leads to the cancer-predisposing genetic disorder ataxia-telangiectasia(A-T) which belongs to genomic instability syndromes [34]. Accumulated evidence has indicated that ATM gene polymorphisms are closely related to lung cancer [35]–[37]. Besides, potentially functional polymorphisms in ATM gene may serve as potential prediction biomarker for cancer [8], We observed the association between rs189037 polymorphism in ATM gene and lung cancer in a Chinese Han population, which provides evidence for rs189037 to be a prediction biomarker of lung cancer. Consequently, based on data from different kinds of SNPs, biosensors could be designed [38] to protect normal tissue from cytotoxic effects in treatment, increasing therapeutic effect of genotoxicity drugs or other treatments.

## Conclusions

In conclusion, our research showed that ATM rs189037 single nucleotide polymorphism was associated with lung cancer risk in Chinese Han population. AA genotype and A allele may increase susceptibility of lung cancer. Our study provided strong evidence to further research of large population in different races. Moreover, the contribution of ATM gene mutations to cancer susceptibility is deemed to a bright lamp of cancer epidemiology.

## Supporting Information

S1 Table Genotype distribution of ATM rs189037 and its associations with lung cancer risk.
(DOCX)

S2 Table Rs189037 genotype distribution and lung cancer risk in smokers.
(DOCX)

S3 Table Rs189037 genotype distribution and lung cancer risk in non-smokers.
(DOCX)

S4 Table Genotype distribution of rs189037 and smoking and lung cancer risk.
(DOCX)

S1 Data Initial data of genotyping results.
(XLS)
