# Supplementary material for: Effect of Single Nucleotide Polymorphism Rs189037 in ATM Gene on Risk of Lung Cancer in Chinese: A Case-Control Study
Source: PLoS One. 2014 Dec 26;9(12):e115845. doi: 10.1371/journal.pone.0115845 (PMC4277362; doi:10.1371/journal.pone.0115845)
Supplement: S1 Table — Genotype distribution of ATM rs189037 and its associations with lung cancer risk. (DOCX) [file pone.0115845.s001.docx]

## Table S1

**Genotype distribution of ATM rs189037 and its associations with lung cancer risk**

|  | Genotype | Case (%) | Control (%) | OR(95%CI) ^a^ | P | Adjusted OR(95%CI) ^b^ | P |
| --- | --- | --- | --- | --- | --- | --- | --- |
| Total population | GG | 217（25.5） | 264（31.0) | 1 |  |  |  |
|  | GA | 435（51.1） | 434(50.9) | 1.22(0.98-1.53) | 0.082 | 1.23(0.98-1.55) | 0.08 |
|  | AA | 200（23.5） | 154(18.1) | 1.58(1.20-2.08) | 0.001^*^ | 1.56(1.18-2.08) | 0.002^*^ |
| male | GG | 124（25.6） | 150（30.6) | 1 |  |  |  |
|  | GA | 256（52.8） | 251(51.2) | 1.23(0.92-1.66) | 0.162 | 1.28(0.95-1.75) | 0.11 |
|  | AA | 105（21.6） | 89(18.2) | 1.43(0.99-2.07) | 0.059 | 1.43(0.97-2.09) | 0.07 |
| female | GG | 93（25.3） | 114（31.5) | 1 |  |  |  |
|  | GA | 179（48.8） | 183(50.6) | 1.20(0.85-1.69) | 0.299 | 1.17(0.82-1.65) | 0.39 |
|  | AA | 95（25.9） | 65(18.0) | 1.79(1.18-2.72) | 0.006^*^ | 1.75(1.14-2.68) | 0.01^*^ |

^a^ OR, odds ratios; CI, confidence interval;

^b^ Adjusted for age, gender, smoking status in total population; Adjusted for age, smoking status in female and male population;

^*^P<0.05
