# Supplementary material for: Effect of Single Nucleotide Polymorphism Rs189037 in ATM Gene on Risk of Lung Cancer in Chinese: A Case-Control Study
Source: PLoS One. 2014 Dec 26;9(12):e115845. doi: 10.1371/journal.pone.0115845 (PMC4277362; doi:10.1371/journal.pone.0115845)
Supplement: S2 Table — Rs189037 genotype distribution and lung cancer risk in smokers. (DOCX) [file pone.0115845.s002.docx]

## Table S2

**Rs189037 genotype distribution and lung cancer risk in smokers**

|  | Genotype | Case (%) | Control (%) | OR(95%CI) ^a^ | P | Adjusted OR(95%CI) ^b^ | P |
| --- | --- | --- | --- | --- | --- | --- | --- |
| Smokers | GG | 120（25.2） | 87（31.9) | 1 |  |  |  |
|  | GA | 249（52.2） | 129(47.3) | 1.40(0.99-1.98) | 0.059 | 1.40(0.99-1.99) | 0.057 |
|  | AA | 108（22.6） | 57(20.9) | 1.37(0.90-2.10) | 0.141 | 1.38(0.90-2.11) | 0.135 |
| Male smokers | GG | 75（25.4） | 55（33.7) | 1 |  |  |  |
|  | GA | 159（53.9） | 72(44.2) | 1.62(1.04-2.53) | 0.034^*^ | 1.63(1.04-2.55) | 0.032^*^ |
|  | AA | 61（20.7） | 36(22.1) | 1.24（0.73-2.13） | 0.43 | 1.25（0.73-2.15） | 0.417 |
| Female smokers | GG | 45（24.7） | 32（29.1) | 1 |  |  |  |
|  | GA | 90（49.5） | 57(51.8) | 1.12(0.64-1.97) | 0.686 | 1.12(0.64-1.97) | 0.69 |
|  | AA | 47（25.8） | 21(19.1) | 1.59(0.80-3.16) | 0.184 | 1.59(0.80-3.16) | 0.185 |

^a^ OR, odds ratio; CI, confidence interval;

^b^ Adjusted for age

^*^P<0.05
