# Supplementary material for: Effect of Single Nucleotide Polymorphism Rs189037 in ATM Gene on Risk of Lung Cancer in Chinese: A Case-Control Study
Source: PLoS One. 2014 Dec 26;9(12):e115845. doi: 10.1371/journal.pone.0115845 (PMC4277362; doi:10.1371/journal.pone.0115845)
Supplement: S3 Table — Rs189037 genotype distribution and lung cancer risk in non-smokers. (DOCX) [file pone.0115845.s003.docx]

## Table S3

**Rs189037 genotype distribution and lung cancer risk in non-smokers**

|  | Genotype | Case(%) | Control(%) | OR(95%CI) ^a^ | P | Adjusted OR(95%CI) ^b^ | P |
| --- | --- | --- | --- | --- | --- | --- | --- |
| Non-smokers | GG | 97（25.9） | 177（30.6) | 1 |  |  |  |
|  | GA | 186（49.6） | 305(52.7) | 1.11(0.82-1.51) | 0.496 | 1.12(0.82-1.53) | 0.465 |
|  | AA | 92（24.5） | 97(16.8) | 1.73(1.19-2.53) | 0.004^*^ | 1.73(1.18-2.52) | 0.005^*^ |
| Male non-smokers | GG | 49（25.8） | 95（29.1) | 1 |  |  |  |
|  | GA | 97（51.1） | 179(54.7) | 1.05(0.69-1.61) | 0.819 | 1.06(0.96-1.62) | 0.79 |
|  | AA | 44（23.2） | 53(16.2) | 1.61(0.95-2.73) | 0.077 | 1.63(0.96-2.76) | 0.072 |
| Female non-smokers | GG | 48（25.9） | 82（32.5) | 1 |  |  |  |
|  | GA | 89（48.1） | 126(50.0) | 1.21(0.77-1.89) | 0.411 | 1.20(0.76-1.87) | 0.435 |
|  | AA | 48（25.9） | 44(17.5) | 1.86(1.08-3.21) | 0.024^*^ | 1.86(1.08-3.21) | 0.025^*^ |

^a^OR, odds ratio; CI , confidence interval;

^b^Adjusted for age

^*^ P<0.05
