# Supplementary material for: Effect of Single Nucleotide Polymorphism Rs189037 in ATM Gene on Risk of Lung Cancer in Chinese: A Case-Control Study
Source: PLoS One. 2014 Dec 26;9(12):e115845. doi: 10.1371/journal.pone.0115845 (PMC4277362; doi:10.1371/journal.pone.0115845)
Supplement: S4 Table — Genotype distribution of rs189037 and smoking and lung cancer risk. (DOCX) [file pone.0115845.s004.docx]

## Table S4

**Genotype distribution of rs189037 and smoking and lung cancer risk**

| ATM | Smoke | Case (%) | Control (%) | OR(95%CI)^a^ | P | Adjusted OR(95%CI)^b^ | P |
| --- | --- | --- | --- | --- | --- | --- | --- |
| GG | - | 97(11.4) | 177(20.8) | 1.0 |  | 1.0 |  |
| GA+AA | - | 278(32.6) | 402(47.2) | 1.26(0.94-1.69) | 0.117 | 1.26(0.94-1.69) | 0.117 |
| GG | + | 120(14.1) | 87(10.2) | 2.52(1.74-3.65) | <0.001^*^ | 2.53(1.75-3.67) | <0.001^*^ |
| GA+AA | + | 357(41.9) | 186(21.8) | 3.50(2.58-4.75) | <0.001^*^ | 3.52(2.59-4.78) | <0.001^*^ |

^a^OR, odds ratio; CI, confidence interval;

^b^Adjusted for age, genders

^*^ P<0.05
